# Association between pachychoroid and long-term treatment outcomes of photodynamic therapy with intravitreal ranibizumab for polypoidal choroidal vasculopathy

**DOI:** 10.1038/s41598-020-65346-w

**Published:** 2020-05-20

**Authors:** Keiko Azuma, Atsushi Okubo, Yoko Nomura, Hanpeng Zhou, Ryo Terao, Yohei Hashimoto, Kimiko Shimizu Asano, Kunihiro Azuma, Tatsuya Inoue, Ryo Obata

**Affiliations:** Department of Ophthalmology, Graduate School of Medicine and Faculty of Medicine, The University of Tokyo, Tokyo, 113-8655 Japan

**Keywords:** Outcomes research, Macular degeneration

## Abstract

We investigated long-term treatment responses in patients with treatment-naïve polypoidal choroidal vasculopathy (PCV) undergoing photodynamic therapy (PDT) with intravitreal ranibizumab (IVR). The medical charts of 14 patients with treatment-naïve PCV who underwent PDT with IVR were retrospectively reviewed. Patients were followed up and treated with additional IVR for ≥3 years. Best-corrected visual acuity (BCVA), central foveal thickness (CFT), greatest linear dimension (GLD) on angiography, polyp regression and central choroidal thickness (CCT) were assessed. Associations between these functional or anatomic outcomes with age, baseline CCT, baseline GLD or choroidal vascular hyperpermeability (CVH) were investigated using univariate and multivariate analysis. Mean logMAR BCVA improved significantly at 3 years (0.34 ± 0.24 to 0.12 ± 0.29, p = 0.003). Greater BCVA improvement and longer time to first recurrence was significantly associated with CVH. Fewer number of IVR retreatment within 3 years was associated with thicker baseline CCT. Mean CCT significantly decreased at 3 years (217 ± 33 µm to 197 ± 48 µm, p = 0.003). Greater decrease of CCT was significantly associated both with greater number of IVR retreatment within 3 years and absence of CVH. These results showed that pachychoroid characteristics at baseline was associated long-term functional and anatomic outcomes in patients with treatment-naïve PCV who had undergone combination PDT and IVR.

## Introduction

Polypoidal choroidal vasculopathy (PCV), a subtype of neovascular age-related macular degeneration (AMD), is characterised by the observation of polypoidal lesions and branching vascular networks (BVNs) on indocyanine green angiography (ICGA)^1^. PCV typically occurs in middle-aged to elderly individuals and accounts for 22.3–54.7% of all cases of neovascular AMD in Japan^2–5^. The natural course of 50% of patients with PCV includes favourable visual outcomes, whereas in the remaining 50% patients, repeated bleeding and leakage result in macular degeneration and severe visual loss^6,7^. The use of verteporfin (Visudyne; Novartis AG, Basel, Switzerland) PDT as a monotherapy was previously frequently used as a treatment for PCV^8–10^; however, it was associated with a high recurrence rate and a risk of complications such as subretinal haemorrhage and macula atrophy^11^. Anti-vascular endothelial growth factor (VEGF) therapy can reduce the subretinal fluid and improve vision, though anti-VEGF therapy is less effective in inducing complete polyp regression^12^. The combination of PDT and anti-VEGF treatment could achieve significant visual improvement and promote a reduction in disease activity. A randomised clinical trial has demonstrated that PDT combined with intravitreal ranibizumab (IVR) for treatment-naïve PCV ensured significantly greater visual improvement with less persistent polyps than ranibizumab monotherapy^13^. Several meta-analyses have suggested that the combination of anti-VEGF therapy and PDT led to better visual outcomes than only PDT monotherapy or anti-VEGF monotherapy within two years^14,15^. However, the efficacy over a longer period of time was controversial^16,17^. Thus, baseline characteristics predicting the long-term outcomes of combination therapy need to be elucidated. In a previous report, PCV were classified into two subtypes on the basis of the presence or absence of feeder/ draining vessels detected by indocyanine-green angiography (ICGA)^18^. Other studies also supported distinct characteristics in PCV using ICGA and fluorescein angiography (FA)^19,20^. Of note, difference in subfoveal choroidal thickness was one of the major factors to characterize these subtypes^18,19^.

Pachychoroid features are also observed in part of PCV patients^21^. In previous reports, choroidal vascular hyperpermeability (CVH) was seen in 10 to 50% of PCV^22,23^. The response of PCV with pachychoroid features to anti-VEGF treatment was thought to be distinct from those without them, though the definitive results remain controvertial^22,24–27^. Otherwise, several reports have suggested good efficacy of the combination of PDT and anti-VEGF therapy for PCV with pachychoroid over a short-term period^23,28^. However, to the best of our knowledge, no studies have reported the long-term outcomes of combination therapy for PCV with or without pachychoroid features.

In the current study, we hypothesised that long-term functional or anatomic outcomes of combination therapy for PCV might be associated with the presence or absence of pachychoroid features and retrospectively investigated the treatment responses in eyes of patients with PCV undergoing PDT combined with intravitreal ranibizumab as initial treatment and followed up for over a period of 3 years, with a focus on its association with pachychoroid changes.

## Results

In total, eyes of 14 patients with PCV were studied in the present research. There were no cases of high myopia. There were no cases who had contraindication for ICGA. Of these, 9 patients were accompanied by CVH and 5 did not. All 14 patients were treatment-naïve and centre-involving PCV. The average follow-up period was 4.9 ± 1.9 years. The baseline characteristics of the patients before treatment are shown in Table 1.

**Table 1:** The baseline characteristics of 14 Japanese PCV patients with or without CVH.

Variables	Total	CVH (+)	CVH (−)	P value
Number of eyes	14	9	5	
Age (mean ± SD, years)	73.2 ± 6.0	74.9 ± 6.1	70.2 ± 4.9	0.17^*^
Male (%)	8 (57%)	6 (67%)	2 (40%)	0.34^†^
BCVA (logMAR)	0.34 ± 0.24	0.34 ± 0.24	0.34 ± 0.27	0.97^*^
CFT (µm)	185 ± 29	210 ± 20	172 ± 23	0.009^*^
CCT (µm)	217 ± 33	235 ± 17	186 ± 33	0.04^*^
GLD (mm)	3.2 ± 0.9	3.2 ± 0.8	2.9 ± 1.0	0.63^*^

Abbreviations: CVH, choroidal vascular hyperpermeability, LogMAR, logarithm of the minimal angle of resolution; CFT, central foveal thickness, CCT, central choroidal thickness; GLD, great linear demention; SD, standard deviation. *t-test, ^†^Chi-squared test.

All patients included in the study were monthly followed in the pro re nata protocol during the follow-up period and the longest interval of visits was 2 months. The BCVA from baseline to year 3 and the anatomic outcomes are shown in Table 2. BCVA significantly improved from baseline at 3 years (0.34 ± 0.24 to 0.12 ± 0.29, p = 0.003, paired-t test). The mean time to first recurrence was 36.0 ± 27.3 months. The number of IVR retreatment for 3 years was 2.9 ± 3.2. All patients showed at least one recurrence during the follow-up period. No subjects underwent PDT retreatment, thermal laser or other intravitreal therapy. The relationship between logMAR change or anatomic outcomes and the five baseline characteristics (age, baseline VA, CCT, GLD and with or without CVH) were analysed (Tables 3–5 and Supplementary Tables S1–S4). Greater BCVA improvement and longer time to first recurrence was significantly associated with presence of CVH (Tables 3 and 4, respectively). Lower number of IVR retreatment over 3 years was associated with CCT (Table 5). None of the other baseline characteristics were significantly associated with dry macula, CFT change, GLD change or polyp regression at 3years (Supplementary Tables S1–S4).

**Table 2:** The BCVA (logMAR) and the anatomic outcomes in treatment-naïve PCV patients who underwent PDT with IVR.

Variables	
No. of eyes	14
LogMAR baseline	0.34 ± 0.24
after 1 year	0.16 ± 0.29
after 2 years	0.09 ± 0.16
after 3 years	0.12 ± 0.29^*^
LogMAR change for 3 years	−0.22 ± 0.2
The mean time to first recurrence (months)	36.0 ± 27.3
The number of IVR retreatment for 3 years (times)	2.9 ± 3.2
Dry macula at 3 years (%)	71
CFT change at 3 years (µm)	−26.8 ± 48.4
GLD change at 3 years (µm)	57 ± 111
Polyp regression at 3 years (%)	86

Abbreviations: CVH, choroidal vascular hyperpermeability, LogMAR, logarithm of the minimal angle of resolution; CFT, central foveal thickness, CCT, central choroidal thickness; GLD, great linear demention, IVR, intravitreal ranibizumab; SD, standard deviation. *p = 0.0034 compared with baseline logMAR (paired-t test).

**Table 3:** Association between the baseline parameters and BCVA improvement at 3 years.

Variables	Univariate analysis		Multivariate analysis		
	R^2^	P value	Coefficient	SE	P value
Age (years)		0.52	NS		
Baseline BCVA (logMAR)		0.32	NS		
CCT (µm)		0.17	NS		
GLD (mm)		0.72	NS		
With CVH	−0.31	0.033	−0.28	0.13	0.059

R^2^ of the variables without statistical significance were not presented.

Abbreviations: CCT, central choroidal thickness; GLD, greatest linear demension; CVH, choroidal vascular hyperpermeability; NS, not selected in the optimal model.

**Table 4:** Association between the baseline parameters and time to first recurrence.

Variables	Univariate analysis		Multivariate analysis		
	R^2^	P value	Coefficient	SE	P value
Age (years)		0.39	NS		
CCT (µm)	0.53	0.003	NS		
GLD (mm)		0.94	NS		
With CVH	0.59	0.0013	21.1	5.1	0.0013

R^2^ of the variables without statistical significance were not presented.

Abbreviations: CCT, central choroidal thickness; GLD, greatest linear demension; CVH, choroidal vascular hyperpermeability; NS, not selected in the optimal model.

**Table 5:** Association between the baseline parameters and the number of IVR retreatment.

Variables	Univariate analysis		Multivariate analysis		
	R^2^	P value	Coefficient	SE	P value
Age (years)		0.07	NS		
CCT (µm)	0.89	<0.001	−0.09	0.01	<0.001
GLD (mm)		0.51	NS		
With CVH	0.59	0.013	NS		

R^2^ of the variables without statistical significance were not presented.

Abbreviations: CCT, central choroidal thickness; GLD, greatest linear demension; CVH, choroidal vascular hyperpermeability; NS, not selected in the optimal model.

**Figure 1:**
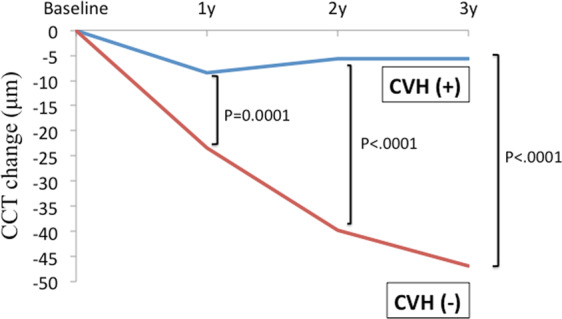
The mean CCT change from baseline at 1, 2 and 3 years in patients with and without CVH. Abbreviations: CCT, central choroidal thickness, CVH, choroidal vascular hyperpermeability.

In total, the mean CCT significantly decreased at year 1, 2 and 3 years compared to baseline (Table 6). The mean CCT change from baseline at 1, 2 and 3 years in patients with and without CVH in Fig. 1. In the multivariate analysis with independent variables of age, CCT at baseline, GLD at baseline, the number of IVR retreatments and CVH, CCT decrease at 3 years was significantly associated with the number of IVR retreatments and CVH; both a greater number of IVR treatments and absence of CVH were associated with a greater decrease in CCT (Table 7). There is no significant association between baseline CCT and mean CCT reduction at month 36 (p = 0.12, Table 7).

**Table 6:** CCT outcomes in patients with PCV with pachychoroid features (N = 9) or those without (N = 5) before and after treatment.

Variables	Total	CVH ( + )	CVH (-)	P value
Baseline CCT	217 ± 33	235 ± 17	185 ± 33	0.0014^†^
CCT at month 12	203 ± 37^*^	226 ± 16	162 ± 27	0.0001^†^
CCT at month 24	199 ± 45^*^	229 ± 13	146 ± 26	<0.0001^†^
CCT at month 36	197 ± 48^*^	229 ± 15	139 ± 22	<0.0001^†^

Abbreviations: CVH, choroidal vascular hyperpermeability; CCT, central choroidal thickness. *p < 0.05 compared with baseline CCT (p = 0.0007, 0.004, and 0.003 at 1, 2, and 3 years, respectively, using paired-t test). ^†^t-test comparing between the patients with CVH and those without CVH.

**Table 7:** Association between the baseline parameters and the CCT decrease for 3 years.

Variables	Univariate analysis	Multivariate analysis			
	R^2^	P value	Coefficient	SE	P value
Age (years)		0.36			
CCT (µm)		0.12			
GLD (mm)		0.54			
The number of IVR	0.23	0.04	3.62	1.10	0.007
With CVH	0.80	<0.001	−29.5	3.50	0.0001

R^2^ of the variables without statistical significance were not presented.

Abbreviations: CCT, central choroidal thickness; GLD, greatest linear demension; CVH, choroidal vascular hyperpermeability; NS, not selected in the optimal model.

## Discussion

In the present study, we investigated treatment responses over a period of 3 years in patients with treatment-naïve PCV undergoing PDT in combination with IVR therapy, especially focusing on the association with pachychoroid features. We found that presence of CVH was associated with greater BCVA improvement at month 36 and longer time-to-first recurrence. Thicker CCT at baseline was associated with fewer number of IVR retreatments within 3 years. The mean CCT was significantly decreased, and its changes were significantly associated with the number of IVR retreatment and CVH. To the best of our knowledge, no report has been published to date on the association between long-term treatment outcomes and pachychoroid features in patients who had received prompt PDT combined with IVR.

The current analyses showed that patients with CVH had their first recurrence after a significantly longer period than those without CVH. Koizumi *et al*.^22,23^ reported that the presence of CVH in PCV patients was significantly related to the persistent retinal fluid 1 month after 3 monthly ranibizumab injections at monthly interval. They suggested that CVH might cause additional exudation due to a non-VEGF mediated pathway, which could lead to suboptimal response to anti-VEGF monotherapy. Meanwhile, good efficacy of the combination of PDT and anti-VEGF therapy for PCV with pachychoroid in a short-term period was reported. Yanagi *et al*. have reported that PCV patients with CVH experienced better visual changes and a fewer number of injections in 1 year after combination therapy in comparison with those without CVH^23^. Another report investigated the outcomes of patients with PCV who underwent combination therapy and were followed up for one year revealed that those who responded to the treatment were more likely to have choroidal hyperpermeability^28^. The reason why PDT is effective in PCV with CVH remains unclear. However, a previous study using OCT angiography showed that a significant reduction in lesion flow or pachyvessels could be observed shortly after PDT was administered for PCV^29^, and a different study suggested that, at one year after PDT, abnormalities in choroidal structure, described as the ratio of the vascular lumen and the stroma, had improved^30^. These prior reports indicate that PDT might improve abnormal choroidal circulation in eyes with CVH by inducing vascular remodelling. Although a large-scale, prospective study will be necessary to demonstrate the hypothesis, the current results imply that the effect of PDT for the eyes with CVH in one year reported by previous studies might be maintained for at least some years. Together these reports suggest that PDT may reduce CVH by inducing vascular remodelling, hence addressing the non-VEGF driven pathogenetic mechanism of this subset of PCV patients.

In the present study, the mean visual acuity significantly improved for 3 years after the combination therapy, and patients with CVH showed better visual improvements than those without CVH. A previous study reported that visual changes tended to be better among patients with CVH at 1 year after combination therapy^23^. Another report revealed that responders to combination therapy, who were more likely to have CVH, gained greater visual improvements at 1 year after combination therapy than non-responders^28^. These results suggested that CVH was one of the associating factors with favourable visual outcomes in a manner that corresponds with better anatomical outcomes. The current study suggested favourable visual outcome, consistent with improved control of disease activity, sustained for at least 3 years by combination therapy.

In the current study, lower number of IVR retreatment over 3 years was associated with thicker CCT at baseline. This result was consistent with a previous report by Sakurada *et al*.^31^ that greater subfoveal choroidal thickness at baseline was associated with less likeliness to require additional treatments in one year after combined PDT with anti-VEGF drugs for PCV. The present result indicated that CCT might also be one of the biomarkers for eyes with pachychoroid features that might be associated with fewer retreatment episodes.

Of note, the mean CCT decreased over a period of 3 years, with a smaller decrease observed in PCV patients with CVH compared to those without. Multivariate analysis revealed that greater CCT decreases were associated with a greater number of IVR treatments and absence of CVH. Considering that retreatment was given via the pro re nata protocol in the present study, having greater number of IVR treatments represented relatively more times of persistent or recurrent exudative changes that occurred during the follow-up period. Once exudative changes occurred, the RPE could be damaged by blood, fibrotic scar or CNV itself. The attenuation of photoreceptors from exudative changes could also induce RPE atrophy^32^. Because the RPE is essential for the maintenance of the choroid^33,34^, RPE atrophy could result in atrophic changes of the choroid. That might be one of the reasons for why the current results showed that a greater number of IVR retreatments was associated with decreased CCT. However, there might be another explanation that repetitive anti-VEGF therapy could possibly cause choroidal thinning, leading in turn to RPE and photoreceptor atrophy. Although a large-scale, prospective study will be necessary to demonstrate the hypothesis, it is natural to be cautious that excessive anti-VEGF suppression might lead to unnecessary choroidal attenuation especially in patients without CVH. On the other hand, the presence of CVH was significantly associated with a smaller decrease in the CCT that was independent of the number of IVR treatments. The mechanism behind this finding is unclear. It is true that PDT was effective for PCV in ameliorating the CNV activity, which resulted in the suppression of the secretion of VEGF from the CNV that could lead to a decrease in the CCT^29^. Moreover, PDT might be also effective in improving choroidal vascular abnormalities that might induce circulatory congestion^29^, resulting in a decrease in the CCT. However, one study suggested that choroidal structures in patients with pachychoroid features might change independently of VEGF^24^. Furthermore, other recent studies suggested that pachychoroid, indicative of choroidal vascular congestion, was attributed, at least in part, to abnormalities in the vortex vein^35,36^. These characteristics of the eyes with pachychoroid changes might be associated with the results of the present study suggesting that the decrease of CCT could occur but might be limited to some extent in comparison with the cases without CVH.

The present study has several limitations. First, this study was a retrospective single-centre study; only a relatively small number of patients were included, which may have led to selection bias. Second, the time when the patients included in the study started the treatment was limited between 2010 and 2011. It was because another anti-VEGF drug (aflibercept) was introduced to the hospital after that period. It led to decline in the number of the patients who underwent combined PDT with IVR, and it was thought that including the patients treated with combined PDT under the condition that aflibercept became another option could cause more biased selection of patients. However, considering that the results in the previous studies^16,17^ were controversial on the long-term effectiveness of PDT, it would be important to investigate the associating factors with the long-term outcomes of PDT even with limited number of patients. Third, criteria for pachychoroid features have not been definitively established. Therefore, the diagnostic criteria for pachychoroid may not have been fully accurate in this research. In future investigations, it may be necessary to accumulate more data to better establish criteria for pachychoroid features. Finally, PCV might have other characteristics that were associated with treatment outcomes of PDT, such as vascular phenotypes revealed by ICGA^18–20^. We could not exclude the possibility that theses vascular phenotypes influenced the outcomes in the current analysis because theses vascular phenotypes were not evaluated in the scope of the present analysis. To elucidate prognostic factors of PDT combined with IVR, a large-scale prospective study should be necessary.

## Conclusion

The presence of CVH, one of pachychoroid features, in PCV was associated with a longer time to recurrence and favourable visual outcomes at 3 years after combination therapy. Clinicians should keep in mind that pachychoroid features such as CVH may be a baseline factor that should be considered in treating patients with PCV.

## Methods

A retrospective study was performed in accordance with the tenets of the Declaration of Helsinki and was approved by the institutional review board (IRB) of the University of Tokyo. All data were fully anonymised before we accessed them. Written informed consent was not required by the IRB but participants who did not grant authorisation for us to use their medical records for the research were excluded from the study.

### Participants

We retrospectively reviewed the medical charts of consecutive patients with PCV who underwent initial PDT combined with IVR from July 2010 to August 2011 in the outpatient clinic of Tokyo University Hospital and were followed-up for at least three years. All patients underwent a standard examination that included the measurement of best-corrected visual acuity (BCVA), slit-lamp biomicroscopy, funduscopy and spectral domain optical coherence tomography (SD-OCT) (Spectralis; Heidelberg Engineering, Heidelberg, Germany) at each visit. BCVA was measured using the Landolt C chart and values were converted into logarithm of minimal angle of resolution (logMAR). Central foveal thickness (CFT) was measured by SD-OCT. The measurement of subfoveal central choroiddal thickness (CCT) was also assessed using SD-OCT images by incorporating enhanced depth imaging OCT images. All patients underwent fluorescein angiography and ICGA at the time of initial treatment unless contraindicated (Heidelberg Retina Angiograph 2; Heidelberg Engineering, Heidelberg, Germany). Greatest linear dimensions (GLD) of the lesions were measured on ICGA images (ICGA-guided GLD), which contained both polypoidal lesions and BVNs. Two investigators (K. A. and Y. N.) independently reviewed the angiography results, determined the presence of polyps and measured the GLD.

### Combination therapy

All patients received combination therapy consisting of a single injection of 0.5 mg IVR, standard-fluence PDT (administered using a 689-nm diode laser unit) (Visulas PDT system 690 S; Carl Zeiss AG, Oberkochen, Germany) following intravenous verteporfin (Visudyne; Novartis AG, Basel, Switzerland) in accordance with the guidelines for applying PDT in PCV^29,37,38^. Additional IVR therapy instead of therapy, as none of the patients received repeat PDT was administered as required in response to exudative OCT findings such as the development or persistence of subretinal fluid, subretinal haemorrhage and active choroidal neovascularisation during monthly follow-up visits.

### Definition of PCV and CVH

The diagnosis of PCV was made based on the criteria of definite PCV defined by the Japanese Study Group of Polypoidal Choroidal Vasculopathy^39^; that is, having protruding orange-red lesions as observed by fundus examination or having characteristic polypoidal lesions as seen in ICGA. ICGA images were used to evaluate CVH. On ICGA, CVH is defined as irregular areas of increasing fluorescence during mid and late phase, often surrounding dilated pachyvessels. Areas of CVH in eyes of patients with PCV were defined by the presence of patchy and multicentric choroidal staining in late-phase ICGA^23^, and these are usually larger than the areas of polypoidal lesions and BVNs. The judgement of CVH from ICGA was made by two experienced retinal specialists (K. A. and Y. N.), and assessed the presence/absence of CVH using late phase ICGA (about 10 minutes). Two independent investigators judged cases in blinded manner, and they reached consensus in all cases.

### Statistical analysis

The baseline characteristics before the provision of treatment in total patients were assessed, and those of the patients with or without CVH were compared using the chi-square test for categorical variables and the t-test for continuous variables. In total, logMAR at baseline and at 3 years were compared using paired-t test. The anatomic outcomes including the time to first recurrence, the number of IVR retreatment for 3 years, proportion of dry macula at 3 years, CFT change at 3 years, GLD change at 3 years, and polyp regression were evaluated. The CCT at baseline and 1, 2 or 3 years were evaluated and compared using paired t-test. The CCT of the patients with or without CVH at baseline, 1, 2 or 3 years were compared using t-test. The relationship between baseline characteristics (age, baseline VA, CCT, GLD and with or without CVH) and the logMAR change or the aforementioned anatomic outcomes were calculated using univariate and multivariate analysis. For the univariate analysis, linear regression models were used for continuous variables, while logistic regression models were used for binary outcomes. If multiple explanatory variables were found significantly associated in the univariate analysis, multiple regression analysis with step-wise selection was performed. First, the explanatory variables were evaluated using a linear model. The optimal linear model was then selected among all possible combinations of predictors, resulting in 2^2^ to 2^4^ patterns based on the second-order bias-corrected Akaike Information Criterion (AICc) index (denotes optimal model)^24^. We used correlation and multiple regression cross-sectional analyses to determine associations of baseline parameters^24^. In the same way, the relationships between the five explanatory variables before and after treatment (age, CCT at baseline, GLD at baseline, the number of IVR treatments and CVH positive or negative) and CCT changes over three years were calculated using a linear model with stepwise selection.

The AIC is a well-known statistical measure used in model selection, and the AICc is a corrected version of the AIC that provides an accurate estimation when the sample size is finite^40^. The degrees of freedom in a multivariate regression model decrease with an increase in the number of variables. It is therefore recommended that clinicians use the model selection method to improve the model fit by removing redundant variables^33,41^.

All statistical analyses were carried out using the JMP version 11.0 software program (SAS Institute, Cary, NC, USA), and P-values < 0.05 were considered to be statistically significant. All values are presented as means ± 1 standard deviations.

## Supplementary information

Supplementary Tables S1–S4.

## Data Availability

The datasets generated and/or analysed during this study are available from the corresponding author upon reasonable request.
