# Supplementary material for: Association between pachychoroid and long-term treatment outcomes of photodynamic therapy with intravitreal ranibizumab for polypoidal choroidal vasculopathy
Source: Sci Rep. 2020 May 20;10:8337. doi: 10.1038/s41598-020-65346-w (PMC7239911; doi:10.1038/s41598-020-65346-w)
Supplement: Supplementary file 1 — Supplementary Tables S1–S4. [file 41598_2020_65346_MOESM1_ESM.pdf]

Association between pachychoroid and long-term treatment outcomes of photodynamic therapy with intravitreal ranibizumab for polypoidal choroidal vasculopathy

Keiko Azuma, Atsushi Ookubo, Yoko Nomura, Hanpeng Zhou, Ryo Terao, Yohei Hashimoto, Kimiko Shimizu Asano, Kunihiro Azuma, Tatsuya Inoue, & Ryo Obata

Supplementary table S1. Association between the baseline parameters and dry macula at 3 years.

Variables	Univariate analysis		Multivariate analysis		
	R^2	P value	Coefficient	SE	P value
**Age (years)**		0.49	NS		
**CCT (µm)**		0.08	NS		
**GLD (mm)**		0.65	NS		
**With CVH**		0.24	NS		

R^2 of the variables without statistical significance were not presented.

Abbreviations: CCT, central choroidal thickness; GLD, greatest linear demension; CVH, choroidal vascular hyperpermeability; NS, not selected in the optimal model. Multivariate analysis was conducted when two or more variables were significant in the univariate analysis.

Supplementary table S2. Association between the baseline parameters and CMT change at 3 years.

**Variables**	**Univariate analysis**		**Multivariate analysis**		
	R^2	P value	Coefficient	SE	P value
**Age (years)**		0.55	NS		
**CCT (µm)**		0.63	NS		
**GLD (mm)**		0.98	NS		
**With CVH**		0.61	NS		

R^2 of the variables without statistical significance were not presented.

Abbreviations: CCT, central choroidal thickness; GLD, greatest linear demension; CVH, choroidal vascular hyperpermeability; NS, not selected in the optimal model. Multivariate analysis was conducted when two or more variables were significant in the univariate analysis.

Supplementary table S3. Association between the baseline parameters and GLD change at 3 years.

Variables	Univariate analysis		Multivariate analysis		
	R^2	P value	Coefficient	SE	P value
**Age (years)**		0.49	NS		
**CCT (µm)**		0.86	NS		
**GLD (mm)**		0.69	NS		
**With CVH**		0.31	NS		

R^2 of the variables without statistical significance were not presented.

Abbreviations: CCT, central choroidal thickness; GLD, greatest linear demension; CVH, choroidal vascular hyperpermeability; NS, not selected in the optimal model. Multivariate analysis was conducted when two or more variables were significant in the univariate analysis.

Supplementary table S4. B Association between the baseline parameters and polyp regression at 3 years.

Variables	Univariate analysis		Multivariate analysis		
	R^2	P value	Coefficient	SE	P value
**Age (years)**		0.17	NS		
**CCT (μm)**		0.94	NS		
**GLD (mm)**		0.21	NS		
**With CVH**		0.60	NS		

R^2 of the variables without statistical significance were not presented.

Abbreviations: CCT, central choroidal thickness; GLD, greatest linear demension; CVH, choroidal vascular hyperpermeability; NS, not selected in the optimal model. Multivariate analysis was conducted when two or more variables were significant in the univariate analysis.
